# Water Resources Carrying Capacity and Circular Economy Based on Fuzzy Multilayer Algorithm

**DOI:** 10.1155/2022/9959933

**Published:** 2022-08-18

**Authors:** Liangyou Ai

**Affiliations:** Digital Economy Academy, Yango University, Fuzhou, Fujian 350015, China

## Abstract

By combining the relevant theoretical foundations such as fuzzy algorithm and water resources environmental management, and selecting the actual water resources integration data, this paper establishes an index system to investigate the carrying capacity of the water environment in this area. Through the study and application of the comprehensive multilevel fuzzy evaluation model, based on scenario analysis, the current situation of water resources environmental management and the temporal and spatial variation characteristics of water resources in the study area in recent years were evaluated. In order to observe the differences more accurately in the spatial structure of water use in the study area through information entropy, ArcGIS IS images were drawn according to the calculation results of the urban degree balance in the study area. In the development of circular economy, the information center plays an important role in the industrial ecosystem, which is the basis for the recycling of materials, energy, and water. By building a unique data platform, it can help companies understand the latest status of logistics, energy, and waste recycling in the park and can make adaptive adjustments to the above conditions, to achieve the sustainable development of the overall industrial chain.

## 1. Introduction

Adequate water resources are a prerequisite for ensuring the social and economic development of a region. With the rapid economic development and the continuous growth of the population, the limited water resources cannot tolerate higher consumption, and the carrying capacity of water resources is facing severe tests [1]. The carrying capacity of water resources is not only an important prerequisite for achieving sustainable development in a region but also the key to solving water resources problems [2]. Through the analysis of the research status of sustainable utilization of coastal water resources at home and abroad, the research on sustainable utilization of coastal water resources mainly focuses on the research on the relationship between supply and demand of coastal water resources and measures the supply and demand of coastal water resources from two aspects. However, there are few studies on the internal role of coastal water resources-socioeconomic-ecological-environment composite system, especially the relationship between water-use efficiency and economic development. With the increasing shortage of coastal water resources, the research on the relationship between water use efficiency and economic development has become the key to realizing the sustainable utilization of coastal water resources; in the selection of evaluation indicators, most studies only focus on the indicators of water supply, water use and social and economic development, while the indicators of ecological environment water use are ignored. With the acceleration of the urbanization process, the impact of human activities on the coastal water resources system has been increasing, and the ecological environment water consumption has gradually increased. Therefore, in the process of considering the sustainable utilization of coastal water resources, the ecological environment water use has become an indispensable factor.

On the basis of analyzing and summarizing the relevant research results of water resources and environmental management at home and abroad, this paper combines the current situation of water resources development and utilization in the study area and cloud computing technology to construct a comprehensive analysis system for water resources in the study area [3]. It uses the process analytic hierarchy process and entropy method to formulate indicator weights and uses a multilevel fuzzy comprehensive analysis model to evaluate and analyze the current status of water resources in the study area and the temporal and spatial changes of water resources [4]. Subsequently, early warning and forecasting of the water resources environmental management status in 2020, 2022, and 2035 were carried out, to propose corresponding measures to improve the water carrying capacity, and to provide a reference for ensuring the rational development, utilization, and distribution of water resources in the study area. Based on this point, the article investigates the pilot projects based on environmental economics [5, 6]. As the main body of the economic zone on the west coast of the Straits, the study area takes marine economy as a strategic force for the future economic society and seeks to establish an economic blue pilot zone on the west coast of the Straits [7]. The key to implementing the development strategy of “Strong Ocean Province” in the research area is to follow the concept of “green growth, circular development and environmental protection” in the process of marine economic development. Research and design a typical model of marine circular economy development, with a view to efficient utilization of marine resources, thereby protecting the marine environment, and correctly handling the interactive relationship between development and environmental protection [8]. From the perspective of industrial development, this article puts forward the basic principles of circular economy development: development purpose, overall structure, industrial concentration, regional structure, and transformation of construction mode, etc. [9]. In the research area's marine economic development model, the research area is focused on marine fisheries (including aquaculture and marine processing industry), marine comprehensive utilization industry, ship dismantling and transformation, coastal tourism, construction, maritime engineering, and shipping [10]. The main ideas for the economic development of the marine environment are based on the above-mentioned industrial resources and environmental advantages and development conditions, to create a customary model of the economic development circle, and finally from the construction and improvement of legal and management systems, policy support systems, technical assistance systems, evaluation index systems, and the advertising and education system put forward opinions and suggestions to promote the development of circular marine economy in the study area [11].

## 2. Materials and Methods

### 2.1. Evaluation Model of Water Resources Carrying Capacity

The basic principle of the fuzzy comprehensive evaluation method is to use the principle of fuzzy linear transformation and the principle of maximum membership degree to quantitatively analyze the indicators of the evaluated things with unclear boundaries and different quantifications. Finally, the evaluation matrix and the weight vector of the factors are fuzzified, and finally the comprehensive evaluation result is obtained. The model is as follows. First, the group *U* composed of different factors is divided into *n* subsets according to its category characteristics, for example:

(1) $$\begin{array}{c} U=\left\{U_{1},U_{2},\ldots ,U_{n}\right\}. \end{array}$$

According to the different levels of the indicator system, construct appropriate valuation indicators, and set factor sets in each set, for example:

(2) $$\begin{array}{c} U_{i}=\left(u_{i1},u_{i2},\ldots ,u_{is}\right),\;i=1,2,\ldots ,s. \end{array}$$

Second, create a set in the first evaluation level of the *B*_*i*_-factor subset and set the evaluation set to:

(3) $$\begin{array}{c} V=\left\{\begin{array}{c} v_{1},v_{2},\ldots ,v_{m} \end{array}\right\}. \end{array}$$

The weight distribution *U* is:

(4) $$\begin{array}{c} W_{i}=\left(w_{1},w_{2},\ldots ,w_{n}\right). \end{array}$$

Here ∑_*j*=1_^*n*^*w*_*ij*_=1, the unique factor matrix of the *U*_*j*_ test is set to *R*_*j*_, and the comprehensive value of the first level test is

(5) $$\begin{array}{c} B_{i}=W_{i}^{o}R_{i}=\left(b_{i1},b_{i2},\ldots ,b_{in}\right),\;i=1,2,\ldots ,s. \end{array}$$

Taking each *U*_*i*_ as an element and *B*_*i*_ as a separate factor of its evaluation, the evaluation matrix *R* is executed as follows:

(6) $$\begin{array}{c} R=\left[\begin{array}{c} B_{1}\\ B_{2}\\ \vdots \\ B_{s} \end{array}\right]=\left[\begin{array}{ccc} b_{11}b_{12} & \ldots & b_{1m}\\ b_{21}b_{22} & \cdots b_{2m} & \;\\ \vdots & \vdots & \vdots \\ b_{s1}b_{s2} & \cdots & b_{sm} \end{array}\right]. \end{array}$$

It is a series of factors used to evaluate {*U*_1_, *U*_2_,…, *U*_*n*_}. The principle is to identify each *U*_*i*_ as a component of *U*, show the specific nature of *U*, and assign weights according to its importance.

(7) $$\begin{array}{c} W=\left(w_{1},w_{2},\ldots ,w_{s}\right). \end{array}$$

Therefore, the secondary assessment is

(8) $$\begin{array}{c} B=W^{\circ }R. \end{array}$$

Common operators are defined as

(9) $$\begin{array}{c} \forall a,b\in \left(0,1\right),a\vee b=\max\left(a,b\right). \end{array}$$

The main factor that determines the model *M*(^, *v*) is the ordinary fuzzy operator, which is the most common type of fuzzy operator. The calculation method is

(10) $$\begin{array}{c} B_{i}=V_{i=1}^{m}\left(W_{i}\wedge R_{ij}\right). \end{array}$$

### 2.2. Grading Standards for Evaluation Indexes of Coastal Water Resources Carrying Capacity

The discharge of unit equipment wastewater is based on the actual situation of the study area, and the average values are selected as 2000 m³/km^2^, 1000 m³/km^2^, 500 m³/km^2^, 300 m³/km^2^.

The vegetation coverage index is not only a prerequisite for water resources circulation but also reflects the regional water and soil conservation and water conservation capabilities. According to the actual situation of the study area, the critical values are 50%, 60%, 70%, and 80%.

The classification of different indicators is shown in Table 1.

### 2.3. Data Collection Method

The main content of this paper is to support and develop the conceptual definition and theoretical basis of the marine circular economy, summarize the research methods of the marine circular economy and create a systematic analysis, summarize the marine development mechanism and model, and establish an evaluation index system for the marine circular economy, taking the study area as an example, geography, industrial ecology, cleaner production, resource recycling theory, industrial agglomeration, productivity distribution, a comprehensive approach to research issues such as different levels of evaluation theory for the marine circular economy. This research should adopt an innovative research method to establish a sensitive and effective model of environmental impact factors. The biggest feature of this method is to design a cloud computing-based IoT platform that can be summarized and operated. The Impact of Environmental Impact on Human Development aims to integrate quantitative environmental impact models to provide a research platform for more accurate exploration of the relationship between marine economic development and environmental protection. It is based on the innovative research perspective and research theory of the next generation of information technology and explores this topic in the quantitative research path. The platform is used to monitor various statistical monitoring parameters of the impact of economic development on the environment. The system is based on the cloud computing IoT architecture and uses relevant detection tools and sensors to detect the attributes of the measurement parameters. Automatic or semi-automatic detection of the continuous data of the target object, the platform automatically and semi-automatically collects and analyzes the data, and then formulates relevant plans for editing according to the monitoring and evaluation of the data by the relevant monitoring models.

Data collection was based on a combination of online literature collection and field research collection. The research process includes a combination of cloud computing methods, data retrieval and online query, data collection monitoring, typical field investigation, and indoor data sorting and analysis. In the process of empirical analysis on the development of marine circular economy in this research field, a combination of qualitative analysis and quantitative analysis is used. Specifically, the system dynamics analysis and cloud computing software are used for modeling, and on this basis, the problems existing in the development of the marine circular economy in the study area are identified. In the evaluation of the development of marine circular economy in this research field, methods such as AHP and weighted average based on actual conditions are used.

## 3. Results

### 3.1. Current Status of Water Resources Development and Utilization

According to the graphs of the water consumption of the three industries, the main industry's water consumption has always been the main body of production water; the primary and secondary industries have experienced many changes before the 21st century and gradually stabilized after entering the 21st century. Water consumption has been at a low level since it was produced.

**Figure 1:**
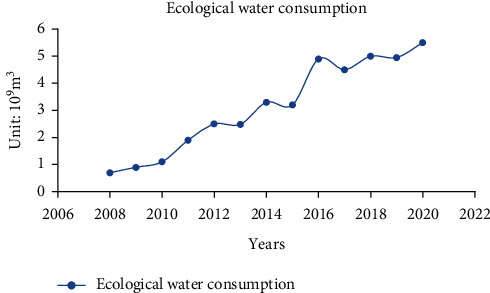
Trend of ecological environment water consumption in the study area.

At the end of the 20th century, with the gradual deterioration of the environment, people began to pay attention to the issue of ecological water use and make records. As a large province in the northeast old industrial base, the research area is particularly important for its industrial development characteristics, adjustment of industrial structure, and coordination of the relationship between industry and ecology. As the ecological environment water consumption was recorded late, the curve diagram is shown in Figure 1 based on the ecological environment water consumption data in the study area from 2008 to 2020.

According to calculations, the average annual growth rate of water consumption in the ecological environment from 2008 to 2020 is as high as 16.58%. It can be seen from Figure 1 that, except for a slight decrease in the water consumption in the ecological environment in individual years, the overall increase has remained relatively large increase. Although the eco-industry is still in its infancy in the research area, with the increasing attention of people and the improvement of environmental protection awareness, the eco-industry has great potential and is also a new type of industry with good market prospects that is worth exploring.

**Figure 2:**
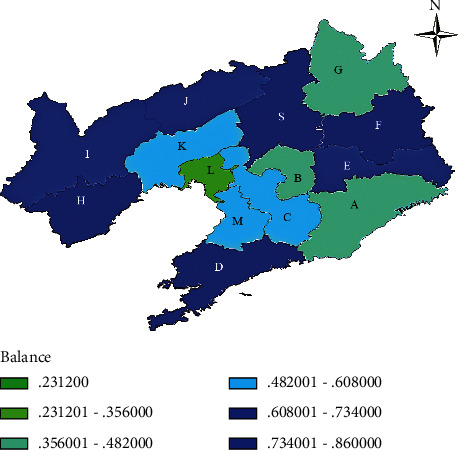
Distribution map of the water structure balance of the cities under the jurisdiction of the study area in 2020.

In order to observe the differences more accurately in the spatial structure of water use in the study area through information entropy, based on the calculation results of the urban degree balance in the study area, the 2020 ArcG IS image is drawn as shown in the following Figure 2.

As shown in Figure 2, by 2020, the color difference of images will increase, so the structural difference of water use in each city will increase. City D in the southern region has the most balanced water use structure and has always maintained the highest level in the province. City S in the northern region has optimized its water use structure, while City A, City B, and City L in the central region are more unstable than in 2010 or even 2005; City H in the western region compared with 2010, the water consumption structure has been further improved, and it is basically the same as City S, City F, and even City D.

**Figure 3:**
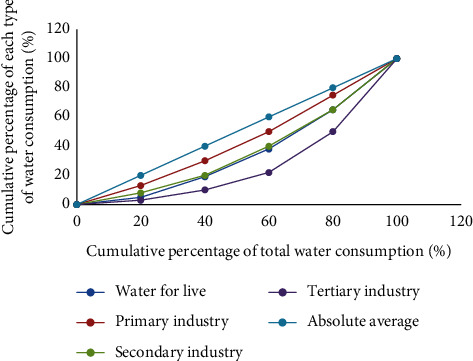
Lorenz curve of water structure in the study area in 2010.

According to the calculation results of the location entropy of each city and the cumulative percentage of domestic and production water consumption in 2010, the Lorentz process curve is shown in Figure 3:

It can be seen from the figure that the minimum curvature curve is the Lorentz curve of the water consumption of the primary industry; the water consumption curve of the secondary industry is the second, and the average is far from the absolute average.

### 3.2. Evaluation Results of Water Resources Carrying Capacity

This article takes 2020 as the research section. Collect project data such as “Research Area Economic Yearbook” and “Research Area Water Resources Bulletin” to calculate and process the original data. The actual values of each indicator are as follows (Table 2).

The calculation of membership function is a key step for comprehensive assessment of water transport capacity.  Table 3 shows the calculation of the membership degree matrix of the 2020 data based on the formula.

The fuzzy comprehensive evaluation makes a comprehensive evaluation of the water resources carrying capacity through a comprehensive evaluation matrix on the basis of considering the interrelationship among various factors affecting the water resources carrying capacity, which makes up for the deficiency of the single factor evaluation. The multilevel fuzzy comprehensive evaluation model is an optimization based on the fuzzy evaluation method. One of its advantages is that it can combine the method of fuzzy mathematics with practice, which reduces the subjectivity of the determination of the index weight and avoids the independence of the bearing factors. The second advantage is that by constructing the membership function, it can make better judgments on multilevel problems, so that the evaluation results are not affected by the set of evaluation objects, etc., which is a relatively scientific and reasonable evaluation method.

### 3.3. Research on the Status Quo of the Development of Circular Economy Industry

According to the economic development of the coastal industrial zone, based on the comparison and selection of waste recycling technology in the sintering process at home and abroad, the specific materials for the steel sintering process and the steel energy recovery method are shown in Table 4. Especially for the recycling of raw materials, the recycling of sintering machine desulfurization by-products, the recycling of sintering ore, and sintering flue gas waste heat, etc.

Information system construction is an important part of the eco-industrial park. The information center plays an important role in the industrial ecosystem, which is the basis for the recycling of materials, energy, and water. By building a unique product marketing platform, it will help companies understand the latest status of logistics, energy, and waste recycling in the park and be able to adapt to the above conditions, so as to achieve the sustainable development of the overall industrial chain. The stable operation of the eco-industrial park is inseparable from supporting infrastructure such as water supply installations, sewage treatment plants, water installations, power supply and heating installations, and sewage treatment. The construction of infrastructure is a necessary condition for realizing efficient recycling management of logistics, energy flow, information flow, and water flow, as well as a prerequisite for ensuring the efficient operation of the park's ecological network.

### 3.4. Analysis of the Ways of Recycling and Utilizing Coastal Resources

**Figure 4:**
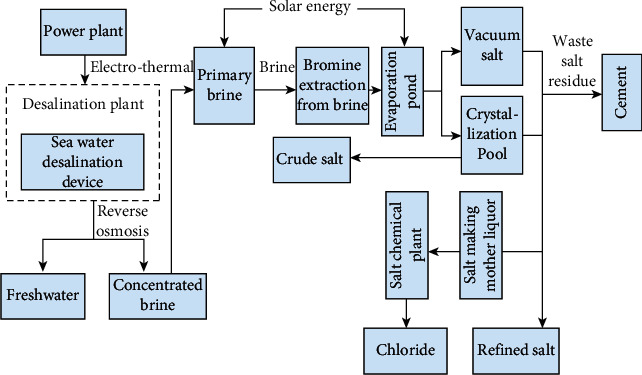
Schematic diagram of the comprehensive utilization of seawater industry chain.

As shown in Figure 4, after the seawater is cooled, its by-products will be discharged in a low-permeability background. The seawater desalination technology produces fresh water and then uses the concentrated salt water produced after reverse osmosis as the raw material of the salt industry to produce potassium, magnesium, bromine, and other salt chemicals to realize the widespread use of seawater.

Solid waste treatment system, the company has strengthened the management of solid waste generated in the process of dismantling ships, the recycling of recyclable parts, and the timely disposal of nonrecyclable parts. Through strict control of recyclable and nonrecyclable solid waste, it can avoid pollution and damage to the environment.

Since the development of this region is greatly affected by policy factors, the relevant indicators of the coastal circular economy are more likely to be affected by administrative intervention. Therefore, this study combines the corresponding planning data and adopts the quota method to analyze the indicators of the coastal circular economy in the study area. Make predictions. This study builds an early warning indicator system for coastal circular economy based on the balance of water supply and demand, which can effectively use the available information and easily reflect the future resource recycling situation. It can be seen from the relevant research results of this paper that the water resources monitoring and early warning system in a certain area can analyze, predict, and evaluate the status quo of the coastal circular economy and the carrying capacity of the future period of time. Warnings will be issued and corresponding control measures will be given.

## 4. Discussion

### 4.1. Construction of a Coastal Circular Economy Model

Based on the development of circular economy in the marine environment, the research area has determined a gradual transition from the traditional linear development model based on the consumption of marine resources to the comprehensive utilization of marine resources in circular development [12]. The marine economic cycle development model is based on the continuous development of the marine industry and the efficient use of marine resources, so as to reduce the harmful effects and damage to the marine environment during the development and utilization of marine resources [13, 14]. By constructing the overall framework of the economic development model of the ocean circle, the lead agency is required to cover enterprises, governments, and the public and promote the development of the ocean economy through their own initiatives.

The core of the marine circular economy circle is the continuous development of the marine industry and the efficient use of marine resources [15]. Based on this, the development of marine recycling industry should focus on marine resource industries such as aquaculture, oil and gas, and comprehensive utilization industries. Since the study area has not yet been involved in the marine oil and gas industry, the development of marine fisheries (including marine aquaculture, product processing, etc.) should be focused on the actual development of the marine industry in the study area [16]. On the other hand, the use, dismantling and construction of marine vessels and coastal tourism, marine engineering construction, and maritime navigation will establish a typical economic environment model based on marine fishing, comprehensive utilization of seawater, dismantling, and reconstruction.

The marine fishery development model mainly introduces the economic development concept of marine aquaculture and seafood processing to realize the reduction, reuse, and recycling of marine resources. The economic development of marine fisheries is an important way to promote the innovation and modernization of traditional fishery development models [17]. From the perspective of the marine fishing industry chain, whether it is the cultivation and fishing of marine biological sources or the processing of marine seafood, it can be reused and updated to conform to the three principles of the economic growth cycle: recycling, reuse, reduction, and circular development, so as to achieve the goal of sustainable growth [18, 19].

**Figure 5:**
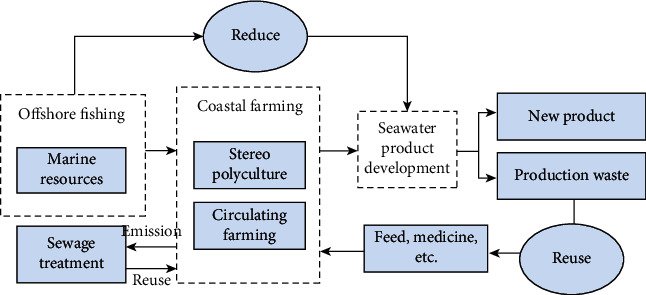
Schematic diagram of the integrated development model of circular marine fisheries.

Creating a comprehensive fishing cycle model, including energy-saving and emission reduction in the breeding process, recycling of seawater, and comprehensive utilization of processing resources, is very important for achieving sustainable growth in marine fishing [20]. Combining the actual situation of the study area, it is possible to consider building an ecological industrial marine fishery park integrating fishing, aquaculture, and seafood processing in a suitable coastal area to form a fishery demonstration base. The standard is consistent with the functions of the natural ecosystem, combining fishing, aquaculture, and marine processing, establishing waste exchange and recycling between industrial waste chains, creating an ecological marine fishery chain, and realizing horizontal symbiosis between industrial industries chain (see Figure 5).

### 4.2. Coastal Water Resources Management Approach

Through the assessment of water resources tolerance in the study area and monitoring and early warning studies, it is found that under the background of open source and restriction, the carrying capacity of water resources has been significantly improved. Therefore, based on the water resources bearing capacity of the study area, the water resources structure was improved, and suggestions based on the four aspects of water resources, economy, society, and ecology were put forward.

Due to the lack of water resources in the study area, the per capita water consumption is low, and the water supply is overreliant on groundwater, the following suggestions are specifically put forward: (1) water storage capacity needs to be improved urgently: forests, grasslands, and wetlands should be expanded gradually and planned, water resources protection and surface runoff coefficient should be improved, and water production should be increased. (2) Strictly control the use of groundwater: strictly control the exploitation of a large amount of groundwater, especially all public wells and self-provided wells and water supply wells in restricted mining areas, prohibited mining areas, and severely overexploited areas. Through closed and comprehensive treatment of groundwater overexploitation areas, groundwater replenishment is strengthened, so as to ensure that the groundwater level stops falling and rises, and finally achieves a balance between groundwater exploitation and filling. (3) Speed up the improvement of water-saving infrastructure: promote the implementation of the second-stage sand source control project in the study area and the national key water and soil conservation project to reduce regional soil erosion and erosion; build rural water supply projects to strengthen rural areas; water and soil loss management and control, implementation of water safety management, and water source protection, etc.

### 4.3. Marine Circular Economy Development Strategy

The development process of circular marine economy is not only a process of innovation and transformation of the existing marine economic development model but also a transformation of the overall model of social progress. In view of the utilization of marine environmental resources, a reliable guarantee system for the economic development of the marine environment will be constructed, so as to provide a good foundation for efficient and stable operation. This article intends to establish and improve the guarantee system for the implementation of the marine economic development model from the perspective of government economic management, including legal and management systems, policy support systems, technical assistance systems, evaluation index systems, and advertising education systems.

Law is the basis for the establishment and implementation of the system, and it is also an effective guarantee for the correct implementation of policies. A sound system of laws and regulations is conducive to creating a fair and just environment for the development of the marine economy and plays a vital role in starting the marine economy of the study area. The direct reason for the change of the model is the introduction of policies to incorporate the development of the marine economy into the macro-level marine economic development plan and to develop a circular economy as the guide, thereby promoting the development of the marine economy. When formulating marine industry policies, the government should focus on improving resource efficiency and protecting the marine environment and promote the restoration and modernization of the marine industry structure. The “Twelfth Five-Year” National Plan clearly proposes to challenge the scientific use of marine resources and actively develop circular economy as an important part of marine economic development. The relevant plans reflect the importance of developing the marine economic circle and promote the development of marine economic policy measures, give play to the leading demonstration role of economic development, play the role of policy guidance and support, and promote the development of marine economy. The policy support system for the development of the marine economic circle in the research area should include the industrial policy system, the fiscal policy system, the tax policy system, and the financial policy system.

In recent years, the study area has maintained good economic and social development with good development opportunities. The main task of government departments at all levels is to promote sustained and rapid economic growth. The economic development of all coastal cities in the study area is currently in a critical period. As the marine economy is still in the rapid development stage, while choosing to continue to develop the marine economic circle and pursue marine economic development, the relevant departments have always focused on the latter, ignoring the traditional economic growth. Therefore, it is necessary to emphasize the importance of government departments in the development of marine circular economy. Relevant departments must strengthen their own construction, establish, and improve marine economic management mechanisms, formulate marine economic policies, and manage and promote the implementation of marine economics in accordance with the law. Strengthen the evaluation and management of government functions, implement policies, and further improve the level of marine economic development. We must pay attention to and solve the problems and needs of different characteristics encountered in the development of marine circular economy and to provide a good development of environment for regional marine circular economy.

## 5. Conclusion

The experimental data prove that the water resources subsystem plays a leading role in the change of water resources carrying capacity in the study area, and the economic subsystem, social subsystem, and ecological subsystem have less influence. However, with the development of social economy, the positive effect of the economic system and the ecological system on the water resources system is becoming more and more obvious, while the social system is putting more and more pressure on the water resources system. In the process of vigorously promoting the development of the development strategy, the problems of marine resources and the environment have become increasingly prominent, and there are practical problems such as the destruction of the marine ecological environment and the waste of marine resources. The development of predatory marine tourism resources has caused serious damage to the marine ecological environment in coastal areas. The development of marine circular economy is of great significance for promoting sustainable utilization of marine resources and reducing marine environmental pollution.

The article investigates the influencing factors of water resources carrying capacity in the study area and integrates the research results of other researchers on the selection of relevant indicators of water resources carrying capacity and selects appropriate indicators from four aspects of water resources, economy, society, and ecological environment. To establish a water source system assessment system in the study area, by dividing the system carrying capacity index and the water carrying capacity state into five categories, the AHP method and the entropy weight method are integrated, the weight index system is combined according to objective factors, and the fuzzy multilevel evaluation method is integrated to select the water resources in the research area in recent years. Finally, based on reading many relevant materials, the author systematically studied various marine industries and related technologies, visited relevant departments, parks, and government-managed enterprises, initially established a typical research model for the development of circular economy, and targeted the goals of the study area. Propose answers to the problems arising in the development process to promote the economic development of the marine environment.

## Figures and Tables

**Table 1:** Grading standards for evaluation indexes of water resources carrying capacity.

Indicator name	Indicator type	*V*_1_	*V*_2_	*V*_3_	*V*_4_	*V*_5_
		Weak	Weaker	Generally	Stronger	Powerful
Water resources per capita	Positive	<311	310–511	511–1000	1000–1700	>1700
Dryness index	Reverse	>6	6–5	5–3	3-2	<2
Water resources development and utilization rate	Reverse	>101	100–80	80–60	60–41	<41
Water production modulus	Positive	<6	6–8	8–16	16–31	>31
Water consumption per 10,000 yuan GDP	Reverse	>101	100–70	70–51	51–41	<41
Water consumption per 10,000 yuan of industrial added value	Reverse	>10	100–51	51–20	20–10	<10
The proportion of tertiary industry	Positive	<31	31–41	41–51	51–70	>70
Irrigation water consumption per mu	Reverse	>411	411–310	310–200	200–100	<100
The population density	Reverse	>411	411–200	200–100	100–51	<51
Urbanization rate	Reverse	>80	80–60	60–41	41–20	<20
Urban water consumption per capita	Reverse	>51	51–41	41–31	31–20	<20
Per capita domestic water consumption in rural areas	Reverse	>60	60–41	41–20	20–10	<10
Ecological water use rate	Positive	<2	2-3	3-4	4–6	>6
Waste water discharge per unit area	Reverse	>2000	2000–1000	1000–511	511–310	<310
Vegetation coverage	Positive	<51	51–60	60–70	70–80	>80

**Table 2:** The actual value of each indicator of the study area's water resources carrying capacity in 2020.

Target layer	Criterion layer	Index layer	Actual value	Unit	Indicator attributes
Water resources carrying capacity	Water resources system	Water resources per capita	297.98	m^3^	Positive
		Dryness index	3.16	None	Reverse
		Water resources development and utilization rate	65.24	%	Reverse
		Water production modulus	3.75	Ten thousand m^3^/km^2^	Positive
	Economic system	Water consumption per 10,000 yuan GDP	63.65	m^3^	Reverse
		Water consumption per 10,000 yuan of industrial added value	20.96	m^3^	Reverse
		The proportion of tertiary industry	48.97	%	Positive
		Irrigation water consumption per mu	175.84	m^3^	Reverse
	Social system	The population density	126.6	%	Reverse
		Urbanization rate	37.96	%	Reverse
		Urban water consumption per capita	38.15	m^3^	Reverse
		Per capita domestic water consumption in rural areas	26.56	m^3^	Reverse
	Ecosystem	Ecological water use rate	1.73	%	Positive
		Wastewater discharge per unit area	164.23	m^3^/km^2^	Reverse
		Vegetation coverage	70.31	%	Positive

**Table 3:** The membership degree of each grade of water resources carrying capacity evaluation index in the study area in 2020.

Criterion layer	Index layer	*V*_1_	*V*_2_	*V*_3_	*V*_4_	*V*_5_
Water resources system	Water resources per capita	1.0000	0.0000	0.0000	0.0000	0.0000
	Dryness index	0.0000	0.0732	0.9268	0.0000	0.0000
	Water resources development and utilization rate	0.0000	0.0000	0.7625	0.2375	0.0000
	Water production modulus	1.0000	0.0000	0.0000	0.0000	0.0000

Economic system	Water consumption per 10,000 yuan GDP	0.0000	0.1828	0.8172	0.0000	0.0000
	Water consumption per 10,000 yuan of industrial added value	0.0000	0.0000	0.5324	0.4674	0.0000
	The proportion of tertiary industry	0.0000	0.0000	0.6044	0.3956	0.0000
	Irrigation water consumption per mu	0.0000	0.0000	0.2584	0.7414	0.0000

Social system	The population density	0.0000	0.0000	0.7651	0.2350	0.0000
	Urbanization rate	0.0000	0.0000	0.3987	0.6014	0.0000
	Urban water consumption per capita	0.0000	0.3176	0.6827	0.0000	0.0000
	Per capita domestic water consumption in rural areas	0.0000	0.0000	0.8286	0.1714	0.0000

Ecosystem	Ecological water use rate	0.0000	0.7800	0.2200	0.0000	0.0000
	Wastewater discharge per unit area	0.0000	0.0000	0.0000	0.0000	1.0000
	Vegetation coverage	0.0000	0.0000	0.4676	0.5324	0.0000

**Table 4:** Analysis of the ways of recycling waste in the sintering process.

Production and waste link	Circular economy technology	Waste, by-products	Where to waste	Circular economy implementation level
Sintering, ironmaking, etc.	Low carbon	Sinter return ore, dust and mud	Reuse for sintering	Enterprise
		Iron oxide scale, steel slag, etc.		
Sintering machine desulfurization	Modified magnesium oxide method	Magnesium sulfate	Used as chemical raw material	Industrial area
Sinter waste heat	Belt ring cooler	Residual heat	External steam supply or power generation	
Sintering flue gas waste heat	Waste heat boiler			

## Data Availability

The data used to support the ﬁndings of this study are available from the corresponding author upon request.
